# Illustrations of the Efficacy of Compression and Percussion in the Cure of Rheumatism and Sprains, Scrofulous Affections of the Joints and Spine, Chronic Pains Arising from a Scrofulous Taint in the Constitution, Lameness, and Loss of Power in the Hands from Gout, Paralytic Debility of the Extremities, General Derangement of the Nervous System; and in Promoting Digestion, with All the Secretions and Excretions

**Published:** 1824-10

**Authors:** William Balfour

**Affiliations:** the University of Edinburgh.


ART. IV.—*Illustrations of the Efficacy of Compression and Percussion in the Cure of Rheumatism and Sprains, Scrofulous Affections of the Joints and Spine, chronic Pains arising from a Scrofulous Taint in the Constitution, Lameness, and Loss of Power in the Hands from Gout, Paralytic Debility of the Extremities, General Derangement of the Nervous System; and in promoting Digestion, with all the Secretions and Excretions.* By WILLIAM BALFOUR, M.D. of the University of Edinburgh.

[Concluded from page 208.]

As the following case was treated by JOHN FOTHERGILL, Esq. surgeon, Darlington, though strictly by my advice and directions, I think it proper to give it in that gentleman's own words:—

CASE XXXVIII.— No. I.

" *From John Fothergill, Esq. to Dr. Balfour.*

" Respected Friend,—In consequence of the favourable impression received from the perusal of thy work on Rheumatism and Gout, by my patient, Thomas Backhouse, of West Lodge, he desires me to transmit to thee the following account of his case; and would be glad to be informed if thou thinkest the principles and remedies detailed in that work would be at all serviceable in his case. A reply as early as convenient will be esteemed a favour.

(Signed) JOHN FOTHERGILL."

" *Darlington, third Month (March)* 13*th*, 1822."

" Thomas Backhouse, now seventy-one years of age, of the sanguineo-nervous temperament, spare habit, and middle stature. From the earliest period of his life has, in general, enjoyed tolerable health; but, for the last thirty-five years, has at times been much troubled with flatulence, accompanied with distressing giddiness; occasionally, a sense of cold and violent shivering; severe spasmodic pains about the chest, shoulders, and back, apparently proceeding from flatus, as eructation generally afforded relief; extreme dimness of sight, which frequently continued for half an hour or more at a time, and sometimes, though not often, was succeeded by violent pain in the head and sickness. He has also suffered from many other affections, generally termed nervous. He has, through the course of his life, been regular and temperate in his manner of living. He has constantly been in the habit of using a good deal of exercise on foot, which he has always thought himself better for. He has not for some years been able to ride on horseback, on account of the giddiness.

" On the 20th, ninth month (September), 1821, he awoke out of a sound sleep with most distressing vertigo, which was much increased by opening his eyes, or on the slightest motion of his head. The bowels have been regulated, throughout the disease, by mercurial alteratives and mild aperients. The alvine discharges have generally exhibited a tolerably healthy appearance, although the secretion of bile has sometimes been vitiated; and. during a severe attack of giddiness, a melænous discharge from the bowels took place, about two months ago, and continued three days; since which it has not recurred.

"The secretions from the kidneys have varied considerably throughout the course of this illness; sometimes being perfectly healthy in appearance, at others loaded with uric acid: and this is uniformly the case when the giddiness is the most troublesome. He often experiences great uneasiness in the bowels, accompanied with flatus; and, during his most severe attacks, it generally escapes by eructation. It is necessary to observe, that, whenever he experiences pain in the abdomen, the extremities are relieved; both parts not being affected at the same time.

"He has now been entirely confined to bed these twenty-five weeks, almost all the time on his back, not being able to move without increasing the giddiness, which is his chief complaint. His appetite has been almost uniformly good throughout this attack. He occasionally complains of pain and stiffness in the muscles of the neck. When the abdomen has been uneasy, he had used friction with advantage."

To the above communication I replied, that, however complicated his complaints, yet that there was a strong gouty diathesis in Mr. Backhouse's case, was very apparent. This view of it, however, did not militate against the adoption of percussion, but rather called for it. Indeed, had I been in search of a case calculated to exhibit the power of this remedy in a striking point of view, I could not have stumbled on or selected a better. Therefore, and as both Mr. Fothergill and his patient seemed fully to comprehend the principles on which percussion proves so powerfully remedial, I gave it as my opinion that it was applicable in Mr. Backhouse's case, with a fair prospect of advantage. Accordingly, I directed it to be applied, under the regulation of the patient's feelings, to the extremities and spine. I also recommended the use of emetic tartar at the same time, as auxiliary to percussion in equalizing the circulation of the blood and distribution of the nervous power. I likewise suggested an issue in the nape of the neck, on account of the overwhelming vertigo.

I conceived my directions were sufficiently explicit; but the case being one of exquisite delicacy, and Mr. Fothergill a stranger (as he candidly acknowledges) to the practice of applying percussion, he hesitated to commence it "without some more particular instructions." Accordingly, I soon received the following communication on the subject.

### No. II.

*From John Fothergill, Esq. to Dr. Balfour.*

"Respected Friend,—I was not favoured with thy obliging reply of the 15th instant, to my letter of the 13th, until this morning, which arose from thy letter having been mis-sent to London. I was afraid, in consequence of the haste in which I was obliged to write my last, that, in the enumeration of symptoms, I might not have given all the requisite information; but, from the conclusions which thou hast drawn, I am persuaded that no material circumstance has been omitted. Thy reference to Case 30* gave me pleasure, as the remarkable effects of percussion on the digestive organs of Lord M. had been pointed out by me to my patient, and led, in a considerable degree, to thy being consulted. My patient feels quite disposed to adopt the mode of treatment which thou hast been so kind to recommend, so far as it is practicable; but it is necessary again to mention, that T. B. always lies on his back, and the giddiness is so urgent on any attempt to alter his position, that at present percussion could not be performed along the spine; and though an issue in the neck would probably have a beneficial effect upon the head, and has frequently been proposed, yet, such would be the difficulty of dressing it, that I scarcely think it can be considered practicable. Even the operation of shaving is frequently attended with much distress. *He has now been six months entirely confined to his bed, and nearly to one posture.* Since I had the pleasure of writing to thee, my patient has considered himself much worse than for some time before.† The lightness in his head, and sensations of disturbance within the abdomen, have been more troublesome. The urine secreted under these symptoms is pale as water, and on cooling deposits a white sediment, although that passed but a few minutes before had been of a clear amber colour, and devoid of sediment; or, at other times, loaded with uric acid. He desires me to inquire, whether percussion of the extremities, the abdominal parietes, and parts to which we can have access, would have such a tendency to relieve some of the most urgent symptoms, by restoring the balance of circulation and nervous influence, as to enable him to change his posture so as to allow of percussion along the course of the spine?

"From continued friction over the surface of the abdomen, Thomas Backhouse has frequently experienced much relief, and the expulsion of great quantities of gas from the stomach and bowels; yet I have no doubt percussion is calculated to prove a much more efficacious stimulus, particularly where there is congestion in deep-seated vessels. At the same time, I am convinced that much will depend on the manner in which it is employed; and I am afraid, *without some more particular instructions*, we should not be able to give the fullest effect to a remedy, of which I have formed considerable expectations.

"With respect to the use of antimonial wine, I would ask whether my patient ought to continue, at the same time, a practice which for years he has considered beneficial to him,—viz. he has taken a little rhubarb and magnesia every day? Would it be better to suspend its use when he is taking tartarised antimony? As he perspires a good deal in health, it is very probable that antimonials are required now, as there has not for some time been much sensible perspiration, although the skin feels soft and healthy. The alterative pills which he has taken have generally contained some preparation of antimony.

---

* See my *Treatise on Rheumatism, &c.* 1816 and 1819.

† I beg the reader to bear in mind that the operation of percussion had not been put in practice at this period.

"When he has the worst attack of giddiness, he cannot bear his eyes open, as the light greatly increases the vertiginous symptoms; but, when he is better, there is no intolerentia lucis: he is able to read a great deal.

"My patient has given a long trial (in addition to the alteratives and aperients mentioned in my last,) to various preparations of colchicum, particularly the acetum colchici, with magnesia and sulphate of magnesia; and also the extract of colchicum, prepared by evaporating the acetum colchici.

(Signed) "JOHN FOTHERGILL."

"*Darlington; third Month (March) 21st,* 1822."

From this second letter of Mr. Fothergill, it appears that Mr. Backhouse was getting worse instead of better; and that he was altogether in a state of such extreme delicacy, that both practitioner and patient were afraid to begin percussion, though they anticipated considerable advantage from it. Imagining myself, therefore, at the bed-side of the patient, I stated that I would begin with percussion at the most distant parts of the extremities, the soles of the feet, in the most gentle manner, but would increase the force as I found the patient could bear it; that I would travel over the extremities and such parts of the pelvis as I could reach (the patient lay on his back), in the same slow and cautious manner; and attempt the spine as soon as circumstances would permit. I assured Mr. Fothergill that, if his patient could bear the weight of a feather falling on him, he need not be afraid to apply percussion, because, though an efficient, it was a perfectly harmless remedy; and that I had no doubt he would find his patient able to bear its application with more freedom, at every succeeding operation. The result of these directions and assurances will appear from the following communication:—

No. III.

*From John Fothergill, Esq. to Dr. Balfour.*

"Respected Friend,—I was duly favoured with thy letter of the 23d ultimo, respecting our friend Thomas Backhouse; and, according to the directions, percussion has been applied twice a-day to the superior and inferior extremities, and to such parts of the spine as can be reached. Friction having been used over the abdomen previous to our correspondence with thee, and apparently with advantage, it has been continued. These applications have been exceedingly grateful to our worthy patient, producing an agreeable sensation, and a more healthy feeling and appearance of the surface.

"His general health is undoubtedly improved in a very considerable degree.

"He has been able, for the last week, to remove from his bed to a couch, where he has remained several hours every afternoon.

"But the vertiginous symptoms occasionally recurring, accompanied with much uneasiness within the abdomen, he remains still a great sufferer from these symptoms.

"My patient has tried repeated small doses of the antimonial wine; but he thought it did not agree with him, and could not be prevailed upon to continue its use.

"Notwithstanding the ample and clear directions with which thou wast so kind as to furnish us for the application of percussion, I cannot for a moment suppose that it has been practised in such a manner as to give the fullest effect to so valuable a remedy; arising partly from our not being adepts in the art, and partly from the circumstances of the case.

"I believe my patient is fully sensible of the great benefit which he has certainly derived from percussion, and I have no doubt he will continue it; but he sometimes fears that it will be inadequate to the removal of the most distressing symptom, vertigo. This, I think, may arise from the imperfect manner in which it is applied, and occasions regret that the distance is so great from Edinburgh, as I should be exceedingly glad if my patient could have the advantage of seeing thee on the spot; but of this I have at present no expectation. This report, however, I think, will be considered, upon the whole, favourable and encouraging. His bowels are regular, with a little assistance from medicine; and the alvine evacuations have a healthy appearance, as also that from the kidneys. The latter, however, is subject to considerable variations, being sometimes pale, particularly when the bowels are uneasy, or the vertigo worse than usual; at others it deposits a white, and occasionally is loaded with pink or lateritious, sediment. If thou wouldst wish any further information on the case, I shall be happy to answer any inquiries, and to hear from thee when convenient.*

(Signed) "JOHN FOTHERGILL."

"*Darlington; fourth Month (April) 25th,* 1822."

Conceiving I had Mr. Backhouse's case fully before me, I was in no hurry to answer Mr. Fothergill's third communication; the more especially, that it was principally a report of the success of the remedy employed. In a few days, however, I received the following from him:—

No. IV.

*From John Fothergill, Esq. to Dr. Balfour.*

"Respected Friend,—I was in hopes of receiving a reply before this to my last letter respecting our patient, Thomas Backhouse; but, not

* I cannot omit this opportunity of saying, that, in a considerable number of cases of rheumatism, sprains, and in several complaints where there has been a disturbed balance of circulation and excitement, I have employed percussion with decided benefit; and particularly in a case of severe spasmodic affection of the hands, fore-arms, and muscles of the right side of the face, with coldness of the extremities, and the pulsation of the radial artery scarcely perceptible; the stomach at the same time so irritable, as to reject food of whatever kind. Being at a few miles distance from my house, I directed percussion to the superior and inferior extremities, and gently along the course of the spine. The spasm soon relaxed, flatus ascended from the stomach, and was succeeded by violent pain in the fore-arm for some time. By proper remedies, the stomach and bowels were soon restored to their usual state.

hearing for some time, I supposed thou mightst be out of town. He desires me to say, that he continues the use of percussion, and his general health improves; but the giddiness is not less troublesome than before.*

"I think I mentioned in my second letter, that T. Backhouse wished, if agreeable to thee, to remunerate thee at the close of our correspondence. In this way he has been in the habit of consulting Dr. Trotter, of Newcastle, and Dr. Scudamore, of London. With the latter he has been in correspondence upwards of four months.†

(Signed) "JOHN FOTHERGILL."

"*Darlington; fifth Month (May) 4th,* 1822."

### No. V.

### *John Fothergill, Esq. to Dr. Balfour.*

"Respected Friend,—I was duly favoured with thy letter of the 7th instant; and am now requested by my patient, Thomas Backhouse, to state that he has been able to leave his bed and his couch, and to sit in the upright position in his arm-chair the whole of the afternoon, for about a week; but certainly with very little diminution of the vertiginous symptoms, the attacks of which are always accompanied with uneasiness within the abdomen. The alvine excretions are regular, and not unhealthy in appearance. The secretion from the kidneys, more especially since T. B. left his bed, has been surcharged with sediment, both red and white. No local evacuation has been practised, nor has the antimonial wine been resumed. It was objected to by my patient, even with the effervescing mixture, from an opinion that medicines in a liquid form have generally been found to disorder his stomach. Percussion has been sedulously employed to all the parts thou didst mention as proper for the application of that remedy,—the soles of the feet, outsides of the inferior extremities, sacrum, ossa innominata, and spine. A few days ago, a slight blackish, or livid, appearanee was observed about the middle of the tibia of each leg: a spirituous lotion was applied, and percussion on those parts avoided. The appearance is subsiding.

"The immediate object of my writing now is to say, that, in consequence of the muscles of the lower extremities having been so long inactive, T. B. is unable to stand without some assistance. He has tried a flannel roller, applied in such a manner as to give moderate and equable support from the foot along the leg, and continued above the knee,

* Mr. Backhouse cannot mean that "the giddiness is not less troublesome" than it was previous to the employment of percussion, because then he was confined to his bed, chiefly to one posture, and had been so for six months; whereas he is stated, in Mr. Fothergill's first report, Letter III. of date 25th April, "to have been able, for the last week, to remove from his bed to a couch, where he remained several hours every afternoon. It follows, therefore, that nothing more is meant, than that "the giddiness was not less troublesome" on the 4th of May than it was on the 25th of April; a circumstance no way incompatible with gradual and general amendment.

† This is another luminous instance of the truth of what I have advanced in my preliminary observations to this series of Cases, that compression and percussion cure many cases of rheumatism, and complaints allied to it, over which, medicine, directed by the most eminent skill, has no power.

which he finds enables him to stand with a great degree of firmness. He would be glad to know if thou thinkest that degree of compression calculated to be useful, and whether any other mode of applying it would be preferable, or likely to afford more assistance. Please to mention the best times for applying the rollers, shouldest thou approve of them; and how often they should be applied, &c.

(Signed) "JOHN FOTHERGILL."

"*Darlington Post-mark*, 23*d May*, 1822."

In answer to this fifth communication, I expressed my unqualified approbation of the bandages to which Mr. Fothergill had had recourse, in order to give support and vigour to his patient's limbs. Compression, indeed, by bandages exclusively, was what I had set out with in my "New Mode of treating Rheumatism and Sprains;" and Mr. Fothergill must have seen many instances of the happy effects of the practice in my "Treatise on Rheumatism," referred to in his first letter. It was the difficulty, or rather impossibility, of applying compression by bandages to many parts which are frequently the seats of rheumatism, that suggested to me the necessity and propriety of making compression with the hands. This method, though not so permanent, might, by repetition, I thought, produce all the effects of permanent compression by bandages. The result justified my anticipations. Again, finding many cases in which the parts affected were so deeply seated as to be unapproachable by compression in any form, I was totally at a loss how to proceed. Reflecting, however, on the effects produced on a sprained limb by the dashing, or falling from a height, of cold water upon it, I concluded that the benefit derived was not from the temperature of the water, but from the concussion produced in the deep-seated and gorged vessels. It occurred to me, therefore, that this process could not only be imitated, but surpassed, by percussion, which might be applied gently or forcibly, so as to affect deep-seated or superficial parts, as circumstances required.

Such were the steps, such the ratiocination, by which I arrived at a method of treating rheumatism and complaints allied to it, which has restored many hundreds to health and strength, who would otherwise have been a burden to themselves and their friends for life; and among these is Thomas Backhouse, Esq. of West Lodge, Darlington, as will appear from the following and last report on his case.

I directed the bandages to be applied with a degree of firmness short of impeding circulation, and only when the patient was out of bed.

No. VI.

*From John Fothergill, Esq. to Dr. Balfour.*

"Respected Friend,—Our patient, T. Backhouse, requests me to hand thee the inclosed ———; and to inform thee that he is considerably better in his general health. He is now able to walk a considerable distance every day, and to ride in his carriage without experiencing giddiness.

"He left home last Friday, in order to spend a few weeks at Seaton.* He has still considerable tenderness of some joints; but, on the whole, it must be considered that his convalescence has been as rapid as could be expected: and I think there is no doubt but percussion has not only tended to relieve the local affections, but to restore the general health. He has continued the use of it up to the present time.

"I remain, very respectfully, thy assured friend,

(Signed) "JOHN FOTHERGILL."

"*Darlington; seventh Month (July) 29th,* 1822."

*Summary.*—Case of a gentleman, aged seventy-one, who had, for the last thirty-five years, been occasionally troubled with distressing giddiness; extreme dimness of sight, succeeded sometimes by violent pain in the head, and sickness; flatulency; a sense of cold and violent shivering; spasms in his breast, back, and shoulders; pain in the bowels and inferior extremities, alternately. In September, 1821, the vertigo suddenly increased to a most distressing degree, and was greatly exasperated by light, or the slightest motion. Henceforth the patient was confined to his bed, chiefly to his back. He had been in this state for six months previous to consulting the author, and was getting worse daily. Cured in about three months, so as to be able to undertake a journey of twenty miles!

---

### Art. V.—*On the After-Pains of Parturition.* By Dr. Kinglake.

* His convalescence is likewise mentioned in a letter from Mr. Thomas Richardson, dated Sunderland, eighth Month (August) 6th, 1822.

